# Antifouling Super Water Absorbent Supramolecular Polymer Hydrogel as an Artificial Vitreous Body

**DOI:** 10.1002/advs.201800711

**Published:** 2018-09-25

**Authors:** Hongbo Wang, Yuanhao Wu, Chunyan Cui, Jianhai Yang, Wenguang Liu

**Affiliations:** ^1^ School of Materials Science and Engineering Tianjin Key Laboratory of Composite and Functional Materials Tianjin University Tianjin 300350 China

**Keywords:** antifouling, hydrogen bonding, supramolecular polymer hydrogels, vitreous body

## Abstract

Recently, there has been a high expectation that high water absorbent hydrogels can be developed as an artificial vitreous body. However, the drawbacks associated with in vivo instability, biofouling, uncontrollable in situ reaction time, and injection‐induced precrosslinked fragmentation preclude their genuine use as vitreous substitutes. Here, a supramolecular binary copolymer hydrogel termed as PNAGA‐PCBAA by copolymerization of *N*‐acryloyl glycinamide (NAGA) and carboxybetaine acrylamide (CBAA) is prepared. This PNAGA‐PCBAA hydrogel physically crosslinked by dual amide hydrogen bonds of NAGA exhibits an ultralow solid content (1.6, 98.4 wt% water content), and shear‐thinning behavior, body temperature extrudability/self‐healability, rapid network recoverability, and very close key parameters (modulus, antifouling/antifibrosis, light transmittance, refractive index, ultrastability) to human vitreous body. It is demonstrated that the hydrogel can be readily injected by a 22G needle into the rabbits' eyes where the gelling network is rapidly recovered. After 16 weeks postoperation, the hydrogel acts as a very stable vitreous substitute without affecting the structure of soft tissues in eye, or eliciting adverse effects. This supramolecular binary copolymer hydrogel finds a broad application in ophthalmic fields as not only a self‐recoverable permanent vitreous substitute, but also transient intraocular filling for prevention of inner tissues in postsurgical eyes.

## 1 Introduction

Vitreous body is transparent jelly‐like soft tissue that not only provides mechanical support to the retina and other surrounding ocular tissues, keeps the light path clear, but also ensures the transport and infiltration of oxygen and nutrients.^1^ The vitreous body can become dysfunctional and thus result in blindness either due to the opacification, physical collapse, and liquefaction or pathological changes such as hemorrhage, proliferative vitreoretinopathy, proliferative diabetic retinopathy, and retinal detachment.^2^, ^3^, ^4^, ^5^ Currently, ophthalmic diseases are rapidly increasing with aging of population. In these cases, total removal and replacement of the vitreous is desired.^6^, ^7^, ^8^ Clinically, air, perfluorocarbon (PFC), and silicone oil are frequently used as vitreous substitute materials. However, they are unstable and have poor biocompatibility or can cause complications such as glaucoma or cataracts.^9^, ^10^, ^11^, ^12^ For example, the air in the vitreous cavity continues to be absorbed, thus losing the ability to support the retina and the surrounding tissue, which eventually leads to retinal detachment. Previous reports have demonstrated that the migration of silicone oil droplets into the retina and the optic nerve caused severe damage to the eyes. Hence, there is an urgent need to develop a long‐term biosafe vitreous substitute.

So far, a variety of natural polymers have been tested as possible vitreous substitutes such as collagen, gelatin, and hyaluronic acid, due to their good biocompatibility. However, they disappeared or diluted in the vitreous cavity soon after implantation because of rapid degradation. Thus they are unsuitable for use as a long‐term vitreous substitute.^10^, ^13^, ^14^ In contrast, synthetic nondegradable polymer hydrogels with cross‐linked 3D network and high water content enable a broad scope of screening as a potential vitreous substitute due to their wide tailorability in mimicking the physicochemical properties of the natural vitreous body. As such, several in situ forming hydrogels have been proposed as vitreous substitutes, including polyethylene glycol (PEG) and polyacrylamide (PAM) hydrogels.^15^, ^16^ In emulating collagen and hyaluronic acid in natural vitreous body, thiol‐containing gellan/poly (methacrylamide‐*co*‐methacrylate) copolymer was designed and synthesized, and injected into vitreous cavity, where a stable reversible hydrogel capable of providing osmotic pressure to reattach the retina, was instantaneously formed upon oxidation. This hydrogel exhibited the optical and rheological properties very close to natural vitreous. However, the residual thiol groups may affect the biocompatibility of the implanted substitute.^17^ A vitreous substitute based on in situ Michael addition reaction between thiol‐modified multifunctional PEG and a vinyl‐containing polymer was shown to exhibit a better space‐filling ability and better biocompatibility.^18^, ^19^ Nevertheless, the resultant gels were not spatially homogeneous, thus leaving room for cell infiltration, and PEG hydrogel was readily degradable in vivo, leaving a concern for its long‐term stability.^20^ Recently, a hydrogel with ultralow polymer content was developed as an artificial vitreous body. This low toxic hydrogel was quickly formed in situ via crosslinking of clusters of highly branched tetra‐armed poly(ethylene glycol) prepolymers whose terminal was modified by thiol and maleic anhydride. The hydrogel did not show any adverse effects in rabbit's vitreous cavity for one year, meanwhile showing a therapeutic and prophylactic effect on retinal detachment.

Recent studies have shown that crosslinked polymer hydrogels in vitro can also be used as vitreous substitutes, but the precrosslinked hydrogels tend to break up into fragments when injected through a small gauge needle, causing an errant light transmission and nutrient transport.^21^, ^22^

**Scheme 1:**
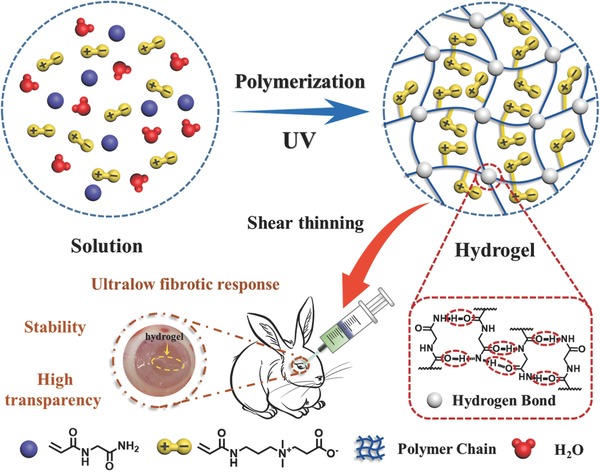
Schematic illustration of the molecular structure of PNAGA‐PCBAA binary copolymer, hydrogen bonding crosslinked network and injection of the formed supramolecular polymer hydrogel via a small gauge needle into rabbit's eyes as a vitreous substitute.

In quest for long‐term vitreous replacements, it has been recognized that apart from high transparency, swelling stability, bioinert, and antifouling ability to inhibit fibrotic response, the substitute should be designed to be structurally self‐adjustable and self‐adaptable in response to in vivo microenvironment to finely fill the space between the lens and the retina of the eyeball. This is reminiscent of supramolecular polymer (SP), a fascinating class of soft materials that are connected by reversible noncovalent interactions and exhibit dynamic properties. In our previous work,^23^ we reported on a high strength, highly stable thermoplastic supramolecular p(*N*‐acryloyl glycinamide) (PNAGA) hydrogel based on strengthening mechanism of dual amide hydrogen bonding in side chains. However, the PNAGA hydrogel was only injectable at a temperature far beyond the physiological range therefore preventing its use in a living environment. In our follow‐up work, we also demonstrated both experimentally and theoretically that the hydrogen bonding interaction intensity could be weakened by copolymerizing with other monomers, thus adjusting the gel–sol temperature to fall in the high biological range.^24^ However, those copolymer hydrogels lacked shear‐thinning behavior at room temperature, network healability at body temperature and remarkably low fibrotic response, which are extremely critical for a vitreous substitute. In light of super antifouling/antifibrosis ability of zwitterionic poly(carboxybetaine) (PCBAA),^25^ in this work, we reported for the first time a poly(*N*‐acryloyl glycinamide‐*co*‐carboxybetaine acrylamide) (PNAGA‐PCBAA) supramolecular copolymer (SP) hydrogel‐based vitreous substitute, which could be injected by shear‐thinning at room temperature through a 22G needle, and quickly gelled in vitreous cavity at body temperature without resorting heating or any chemical reaction (**Scheme** **1**). We will demonstrate that this SP hydrogel artificial vitreous body can maintain high transparency, stability, self‐adjustability, and ultralow fibrotic response in recovering its biological functions.

## 2 Results and Discussion

### 2.1 Characterizations of PNAGA‐PCBAA Supramolecular Polymer Hydrogels

In this work, in order to construct a highly stable and antifouling vitreous substitute, we prepared PNAGA‐PCBAA hydrogels through photoinitiated copolymerization. The molecular and network structure was depicted in Scheme 1. The ^1^H NMR spectra presented the characteristic peaks of NAGA and CBAA monomer units, suggesting the formation of copolymer (Figures S1 and S2, Supporting Information). We prepared a series of aqueous solutions of NAGA and CBAA, which were subjected to photoinitiation. We found that the gelation occurred when the initial concentration of monomers was above 3 wt% (Figure S3, Supporting Information). One interesting phenomenon is that CBAA monomer powder could absorb moisture from air and turn into droplets within 30 min (Figure S4, Supporting Information), assuming a hygroscopic characteristic due to the superhydrophilic nature of the CBAA monomer. This excellent water holding ability will be greatly beneficial for ophthalmic transplantation. It was noted that PNAGA hydrogel and PNAGA‐PCBAA hydrogel could be dissolved in 5 m of sodium sulfocyanate solution, a hydrogen bonding breaking agent, indicting physical crosslinkage of dual amide hydrogen bonding existent in the hydrogel network.^26^

### 2.2 Viscoelasticity and Self‐Healability of the Hydrogels

Rheology is an effective technique for characterizing the viscoelasticity of supramolecular hydrogel materials, offering a criterion to judge whether the hydrogel is suitable for injecting directly into vitreous cavity. In this study, the rheological properties of the PNAGA‐PCBAA‐b‐4 (*b* = 10, 15, and 20) hydrogels were measured.

**Figure 1:**
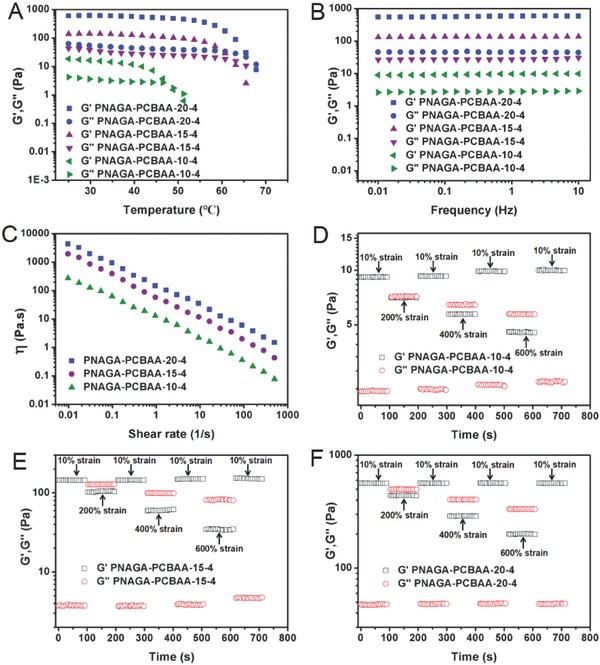
Rheology test of PNAGA‐PCBAA‐10‐4, PNAGA‐PCBAA‐15‐4, and PNAGA‐PCBAA‐20‐4 hydrogels. Variation of dynamic storage moduli *G′* and loss moduli *G′′* in A) a temperature amplitude sweep test from 25 to 70 °C and in B) a frequency sweep test from 0.01 to 10 Hz at 37 °C. Viscosity measurement of PNAGA‐PCBAA‐b‐4 (*b* = 10, 15, and 20) at 37 °C in C) a shear rate sweep from 0.01 to 500 s^−1^. The variation in *G′* and *G′′* values of PNAGA‐PCBAA‐b‐4 (*b* = 10, 15, and 20) hydrogels measured with an alternate step strain being switched from small strain (γ = 10%) to large strain (γ = 200%, 400%, and 600%) at D–F) a fixed angular frequency (1 Hz). Each strain interval was kept as 100 s.

A dynamic temperature sweep rheological test was performed to evaluate the change in the storage modulus *G′* and the loss modulus *G′′* of PNAGA‐PCBAA‐20‐4, PNAGA‐PCBAA‐15‐4 and PNAGA‐PCBAA‐10‐4 over a range from 25 to 70 °C (**Figure** **1**A). Overall, the values of *G′* and *G′′* gradually decreased with increasing temperature. Comparatively, the *G′* and *G′′* values of PNAGA‐PCBAA‐20‐4 hydrogel were higher than those of other two hydrogels due to the stronger hydrogen bonding interaction in the copolymer network at a higher monomer content. It was shown that there were intersection points between *G′* and *G′′* curves of the three hydrogels at about 69, 63, and 47 °C, respectively, depending on the monomer concentration. This implied that gel‐to‐sol transition occurred.^24^ With increasing the monomer concentration, the transition temperature was elevated for the strengthened hydrogen bond interactions. Vial inversion test also demonstrated that heating and cooling treatment led to a reversible sol–gel and gel–sol transition, as shown in Figure S5 (Supporting Information).

As a vitreous substitute, the mechanical properties of a hydrogel are important for fulfilling its supporting function. Next, the frequency sweep mode of rheological measurement was performed on the PNAGA‐PCBAA‐b‐4 (*b* = 10, 15, and 20) hydrogels (Figure 1B). Generally speaking, *G′* was larger than *G′′* at all frequencies from 0.01 to 10 Hz, and there was no crossover, suggesting a “gel‐like” behavior. We noted that the curves of *G′* and *G′′* were almost parallel. This behavior was a typical feature of type IV gel,^27^ suggesting that the PNAGA‐PCBAA hydrogel was suitable for vitreous substitution. As expected, the *G′* and *G′′* values of PNAGA‐PCBAA hydrogels were gradually increased with an increment of the initial monomer concentrations. The *G′* values of the selected three hydrogels varied from 9 to 580 Pa, and the *G′* value of PNAGA‐PCBAA‐10‐4 fell in the range of vitreous body (1–10 Pa).^11^

Another basic requirement for intraocular vitreous injection is the extrudability of hydrogels. Figure 1C shows that the viscosity of the PNAGA‐PCBAA‐b‐4 (*b* = 10, 15, and 20) hydrogels determined at 37 °C decreased markedly with increasing shear rate, demonstrating a shear‐thinning behavior. This suggested that the PNAGA‐PCBAA‐20‐4, PNAGA‐PCBAA‐15‐4, and PNAGA‐PCBAA‐10‐4 hydrogels could be extruded out from the syringe needle by pushing a plunger. It was also observed that the viscosities of PNAGA‐PCBAA hydrogels became higher with increasing the initial monomer concentrations across the entire range of shear rates tested, owing to the denser hydrogen bonding interactions.

The mechanical strength restorability of the hydrogels would determine its feasibility as a vitreous substitute with a direct injection method. The continuous amplitude step strain measurements were performed to test the rheology recovery behavior of PNAGA‐PCBAA‐b‐4 (*b* = 10, 15, and 20) hydrogels. Initially, the PNAGA‐PCBAA‐b‐4 (*b* = 10, 15, and 20) hydrogels was subjected to a small amplitude oscillatory shear strain (10%) at frequency of 1 Hz. In this case, *G′* values were always much greater than the *G′′*, and both the moduli remained invariant with time, implying the solid‐like and elastic nature of the supramolecular polymer hydrogels.^28^ Then, the oscillatory shear strain was increased from 10% to 200%; this change caused an overlap of the *G′* and *G′′*, suggesting the disruption of the hydrogen bonded crosslinking. Notably, both the *G′* and *G′′* quickly recovered their original values after the strain went back to 10% (Figure 1D–F). This hinted that the rapid reconstruction of hydrogen bonding resulted in the complete recovery of the hydrogel network, confirming the excellent recovery capability of the hydrogels in response to the variation from a larger shear strain to a small shear strain. Similarly, when the larger strains (400% and 600%) and small strain (10%) were alternatively exerted later, the *G′* was also observed to recover the initial value after switching back to 10% strain, manifesting that the gel‐like character could be well recovered even subjecting to a larger strain.^29^ These results verified that the supramolecular polymer networks reported in this work exhibited a rapid recovery ability when subjected to alternate shear strain, ruling out the occurrence of irreversible damage of network after undergoing injection. We demonstrated that the PNAGA‐PCBAA‐10‐4 hydrogel could be injected through a 22G needle and quickly gelled upon dropping down to the culture dish at room temperature without heating treatment (Movie S1 and Figure S6, Supporting Information). This characteristic would be beneficial for vitreous substitution without scorching the eye tissues.

It was predictable that the PNAGA‐PCBAA hydrogels might possess the self‐healability on account of their recoverable networks resulted from the reversible reconstruction of hydrogen bonds. Herein, the PNAGA‐PCBAA‐10‐4 was chosen as a representative hydrogel for repairing evaluation. As presented in Figure S7 (Supporting Information), the gel disk was cut into two parts, and then the two semicircles were brought into contact with each other. After incubating at 37 °C for 20 min, the separated semicircles healed completely, and the mended hydrogel disk could bear its own weight and withstand stretching deformation. In our previous work,^23^ we reported that the poly(*N*‐acryloyl glycinamide) homopolymer hydrogels was only repairable when they were subjected to 90 °C high temperature treatment. This harsh condition severely limited their in vivo application. However, for PNAGA‐PCBAA hydrogels, incorporation of zwitterionic units achieved self‐healing in real physiological condition, portending their great potential as a repairable vitreous substitute.

The equilibrium water content (EWC) and optical properties of hydrogels are the key factors to be considered as vitreous substitutes. The vitreous of human adults has a refractive index (RI) of 1.3345–1.3348, 98–99 wt% water content and >90% light transmittance (LT).^30^ The PNAGA hydrogel was shown to have 94.7% water content, 1.3572 RI, but as low as 53.3% and 51.1% light transparency before and 6 months postimplantation, respectively, an indication of translucent property. In contrast, the EWC, RI, and LT of the PNAGA‐PCBAA‐10‐4 hydrogel were very close to those of human vitreous body (Table S1, Supporting Information). After implanted in vivo for 6 months (Table S1, Supporting Information), the PNAGA‐PCBAA‐10‐4 exhibited 92.9% transmittance, indicating proper carboxybetaine modification could contribute to stably high transparency in vivo. Nevertheless, further increasing the initial monomer concentration led to an decreased EWC and LT of the PNAGA‐PCBAA‐15‐4, which deviated from the standard of human vitreous body. We also made a comparison of key parameters between PNAGA‐PCBAA‐10‐4 hydrogel with various hydrogels as vitreous substitutes reported so far (Table S2, Supporting Information). Remarkably, PNAGA‐PCBAA‐10‐4 demonstrated the most close properties to human vitreous body without requiring any reaction condition, meanwhile outperforming all the reported hydrogel‐based vitreous substitutes both in performance and administration. According to the above analysis, the PNAGA‐PCBAA‐10‐4 hydrogel would be chosen as a vitreous substitute candidate.

### 2.3 Antifouling Capability and In Vivo Biocompatibility of the Hydrogels

**Figure 2:**
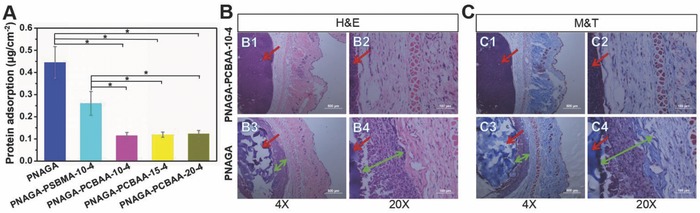
A) Protein adsorption of PNAGA hydrogel, PNAGA‐PSBMA‐10‐4 hydrogel, and PNAGA‐PCBAA‐b‐4 (*b* = 10, 15, and 20) hydrogels. PNAGA‐PCBAA hydrogels exhibited significantly lower protein adsorption compared to PNAGA hydrogel and PNAGA‐PSBMA‐10‐4 hydrogel (p* < 0.05), and no significant difference was observed among the PNAGA‐PCBAA hydrogels. B,C) Micrographs of histological sections stained with H&E and M&T after 1 month implantation of PNAGA hydrogel and PNAGA‐PCBAA‐10‐4 hydrogel. Hydrogels were located on the left side of the images which were denoted by red arrows. B3,B4) Inflammation regions and C3,C4) fibrosis regions were indicated by green arrows. The scale bars are 500 µm (4×) and 100 µm (20×).

The major reason for the failure of the implantable biodevice is the occurrence of biofouling, which has been recognized to elicit the rejection reaction and notorious fibrosis.^31^, ^32^, ^33^ While biofouling usually starts with the nonspecific adsorption of proteins to the implanted biomaterial surface.^34^, ^35^, ^36^, ^37^ In the following study, we evaluated the antifouling capacity of our PNAGA‐PCBAA hydrogels by detecting the protein adsorption. As presented in **Figure** **2**A, the pristine PNAGA hydrogel exhibited ≈0.45 µg cm^−2^ protein adsorption. However, the amount of adsorbed protein on the PNAGA‐PCBAA hydrogels significantly decreased due to the introduction of superior antifouling PCBAA.^38^ In particular, the PNAGA‐PCBAA‐10‐4 hydrogels had only 0.12 µg cm^−2^ protein adsorption. One can find that the protein adsorption was merely slightly enhanced with increasing monomer concentration. It is not difficult to understand that the relative proportion of CBAA in the hydrogel was reduced in this case, thus lowering the antifouling ability. In this study, we also compared the antifouling behavior of the PNAGA‐PCBAA hydrogel with polysulfobetaine‐modified PNAGA hydrogel (PNAGA‐PSBMA) (Figure 2A). The amount of nonspecific protein adsorption of PNAGA‐PSBMA‐10‐4 hydrogel is ≈0.26 µg cm^−2^, which is higher than that of the PNAGA‐PCBAA hydrogels. It is evident that introduction of carboxybetaine zwitterion led to a more pronounced antifouling property of vitreous substitute.

MTT and live–dead staining assays were then performed to evaluate the cytotoxicity of the hydrogels (Figures S8 and S9, Supporting Information). The cell viability of PNAGA and PNAGA‐PCBAA‐10‐4 hydrogels was determined to be 87% and 92%, respectively, indicating better cytocompatibility. Particularly, the PNAGA‐PCBAA‐10‐4 hydrogels exhibited a very low cytotoxicity.

To further examine their biocompatibility, the PNAGA hydrogel and PNAGA‐PCBAA‐10‐4 hydrogel were subcutaneously implanted into the C57BL/6 mice. Histological sections staining with H&E after one month implantation were shown in Figure 2B. For the pristine PNAGA hydrogel, there existed a great number of inflammatory cells surrounding the PNAGA implanted site one month post implantation. Whereas only a mild inflammatory response was detected around the PNAGA‐PCBAA‐10‐4 hydrogel, which were surrounded by much fewer inflammatory cells. The result indicated that PNAGA‐PCBAA‐10‐4 hydrogel had a better biocompatibility.

Figure 2C revealed that the density of fibrosis or deposited collagen stained in blue color was significantly different between the PNAGA‐PCBAA‐10‐4 hydrogel and PNAGA hydrogels. For the PNAGA‐PCBAA‐10‐4 hydrogel, the fibrotic response was much lower compared to the PNAGA hydrogel owing to the superhydrophilicity of PCBAA introduced.^25^, ^39^ This excellent antiprotein property will allow for the alleviating the foreign body reactions. Furthermore, carboxybetaine in CBAA is structurally similar to glycine betaine, one of the critical solutes for regulating the osmosis of living organisms.^25^ Taken together, these endow the PNAGA‐PCBAA‐10‐4 hydrogel with lower foreign body reaction.

As the vitreous substitute, the stability of a hydrogel determined its lifetime. We found that the volumes of PNAGA‐PCBAA hydrogels and PNAGA hydrogel remained constant during immersion in PBS for a long time (data not shown). The stability and excellent biocompatibility of the PNAGA‐PCBAA hydrogels would make it an ideal long‐term vitreous substitute.

### 2.4 In Vivo Evaluation of the Vitreous Substitute

The biocompatibility and very close physicochemical properties to human vitreous body encouraged us to explore the PNAGA‐PCBAA hydrogel as vitreous substitute. On the basis of above analyses, the PNAGA‐PCBAA‐10‐4 hydrogel was chosen and implanted into the vitreous cavity of rabbits. The pristine PNAGA hydrogel served as a control to highlight the pivotal role of PCBAA in this vitreous substitute. Movie S2 and Figure S10 (Supporting Information) show the vitrectomy and injection process of PNAGA‐PCBAA‐10‐4 hydrogel in vivo.

**Figure 3:**
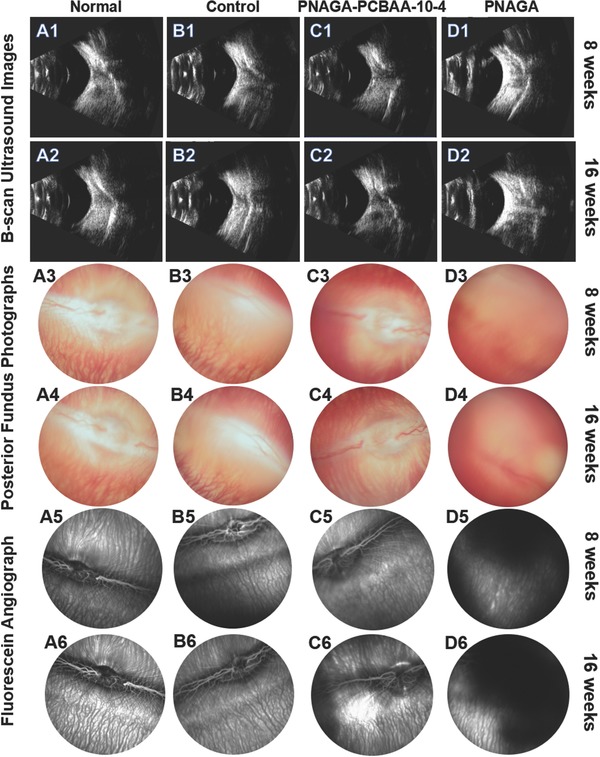
B‐scan ultrasound images, postoperative posterior fundus photographs, and fluorescein angiographs of the four groups' eyes at 8 weeks and 16 weeks post operation. A1–A6) Normal rabbit eyes. B1–B6) Sham‐operated groups. C1–C6) PNAGA‐PCBAA‐10‐4 hydrogel groups. D1–D6) PNAGA hydrogel groups.

B‐scan ultrasound test was a simple and efficient method to examine the retinal integrity. As shown in **Figure** **3**A1–C1, A2–C2, no evident echoes were observed in the normal, sham‐operated and PNAGA‐PCBAA‐10‐4 groups. While for the PNAGA hydrogel group, there were echoes of the unidentified object, which could be originated from retinal detachment or intraocular foreign body reaction elicited by this pristine gel (Figure 3D1,D2).

To further inspect postoperative outcome of the vitreous substitute, funduscopic examination was conducted. For normal, sham‐operated and PNAGA‐PCBAA‐10‐4 hydrogel group, no vitritis, uveitis, retinitis, endophthalmitis, vitreous hemorrhage, or retinal detachment was observed in the rabbit eyes. Blood capillary and central blood vessels were clearly observed, as shown in Figure 3A3–C3,A4–C4. However, no unambiguous vessels and tissue structure could be detected for PNAGA hydrogel group after postoperative 8 and 16 weeks (Figure 3D3,D4). The reasons might be intraocular inflammatory response, vitreous hemorrhage, retina detachment, or low transmittance of PNAGA hydrogel. Fluorescein angiograph measurement further testified that the retina tissue was destroyed in PNAGA hydrogel group. After injecting fluorescent contrast agent, the vessels could be labeled soon, and the contrast medium was dark. For PNAGA hydrogel group, fuzzy and ill‐defined vessels could be found. Abundant contrast agent could be observed in the central of field (Figure 3D5,D6). This could be originated from the neovascularization, and it is most likely that the destruction of the retina led to the leakage of contrast medium. For PNAGA‐PCBAA‐10‐4 hydrogel group, the vessels were clear and distinct. There was nearly no difference among the normal, sham‐operated and PNAGA‐PCBAA‐10‐4 hydrogel groups (Figure 3A5–C5,A6–C6).

**Figure 4:**
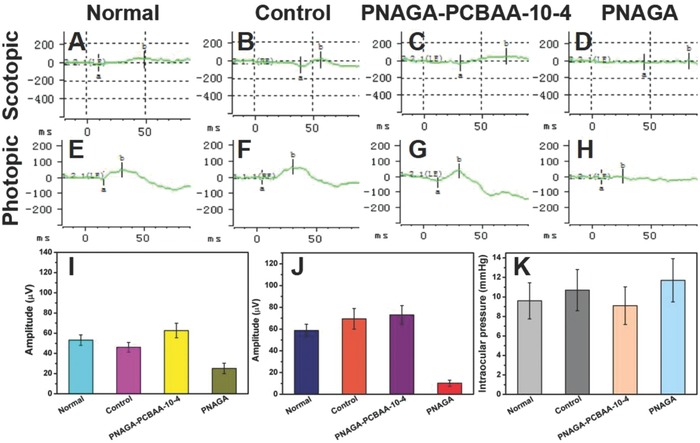
Photographs of postoperative electroretinogram (ERG) and intraocular pressure at 16 weeks. A–D) photographs were scotopic ERG and b‐wave amplitudes recorded from I) graph. E–H) photographs were photopic ERG and b‐wave amplitudes recorded from J) graph. K) The intraocular pressure of various groups at 16 weeks.

Electroretinogram (ERG) measurement was further taken to examine the response of the retina to dark and light conditions. The amplitudes of b‐wave in dark‐adapted ERG and light‐adapted ERG were recorded in **Figure** **4**I,J, respectively. As presented in the figures, there was no obvious difference between the normal group and PNAGA‐PCBAA‐10‐4 hydrogel group. However, in the PNAGA hydrogel‐implanted eyes, the b‐wave amplitudes were significantly decreased in scotopic ERG and photopic ERG during the observation period. A possible reason was that the impaired retina was not healed. Figure 4K shows that the intraocular pressures of the sham‐operated group and PNAGA were higher than that of normal group. Particularly, the PNAGA hydrogel exhibited a much higher intraocular pressure.

**Figure 5:**
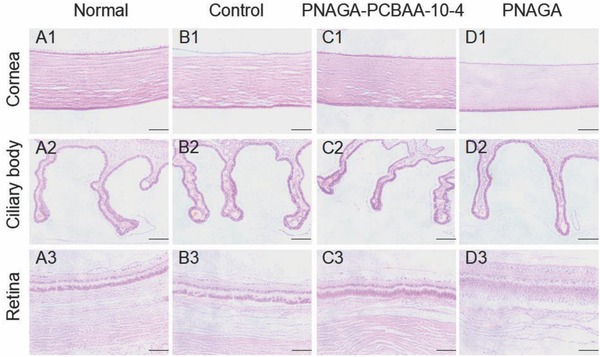
Hematoxylin‐eosin staining of the normal eye and the surgical eyes at 16 weeks post operation. The scale bar of cornea and ciliary body was 100 µm. The scale bar of retina was 50 µm.

**Figure 6:**
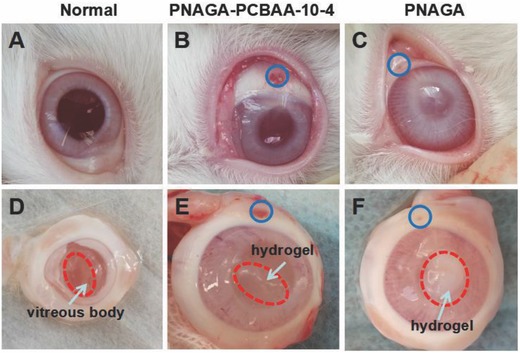
Appearance of A) normal group eye, the eye implanted with B) PNAGA‐10‐4 hydrogel, and C) the eye implanted with PNAGA hydrogel. Incised eyes showing the states of D) normal vitreous body, E) PNAGA‐PCBAA‐10‐4 hydrogel, and F) PNAGA hydrogel implanted in the vitreous cavity for 4 weeks. Blue circle indicated the injection hole.

The histopathologic sections stained with H&E 16 weeks post implantation were examined for checking the eyeball tissues (**Figure** **5**). Histology examination revealed the normal structure and cell morphology of the cornea, ciliary body, and retina in the normal, sham‐operated and PNAGA‐PCBAA‐10‐4 groups at 16 weeks. For PNAGA‐PCBAA‐10‐4 hydrogel group, the structures of cornea and the ciliary body were clearer and more intact. However, the structure of the retina of PNAGA hydrogel group was defective and dissociated (Figure 5D3). And the structure of cornea was a little blurry. **Figure** **6** presents the appearance of eyes and states of the hydrogels implanted in vitreous cavity for 4 weeks. Clearly, like normal vitreous body, the PNAGA‐PCBAA‐10‐4 hydrogel remained stable and very transparent; however, the PNAGA hydrogel became translucent. We noted that the PNAGA‐PCBAA‐10‐4 hydrogel retained high clarity in the designed experimental time frame. Collectively, this polycarboxybetaine modified hydrogen bonding supramolecular polymer hydrogel with the most approximate performance to human vitreous body functioned best as a vitreous substitute.

## 3 Conclusions

In summary, a supramolecular binary copolymer (SBCP) hydrogel composed of hydrogen bonding monomer unit, *N*‐acryloyl glycinamide (NAGA) and zwitterionic monomer unit, carboxybetaine acrylamide (CBAA) was synthesized with aim to address the critical obstacles to the clinical application of high water absorbent hydrogels as artificial vitreous body. We demonstrated that the SBCP hydrogels could hold 98.4% water content and maintain swelling stability by tuning monomer ratios. Apart from shear‐thinning behavior, body temperature extrudability/self‐healability, rapid network recoverability, this SBCP hydrogel is very approximate to human vitreous body in key parameters including modulus, antifouling/antifibrosis, light transmittance, refractive index, which has never been reported before. These attractive physicochemical properties allowed for easy in vivo administration whereby the hydrogel was readily injected at room temperature via a small gauge needle into the rabbits' vitreous cavity where the intact gelling network was rapidly recovered due to the reversible reconstruction of hydrogen bonds. Animal test of the implanted hydrogel 16 weeks postoperation revealed that the hydrogel functioned as an ideal vitreous substitute without eliciting adverse effects. The strategy reported in this study on fabricating supramolecular binary copolymer hydrogel‐based vitreous substitute finds an inspiring approach to design and expand the hydrogels with ultrahigh water content to a broad biomedical application as soft‐wet infill biomaterials.

## 4 Experimental Section

*Materials*: Glycinamide hydrochloride (98%, Tokyo Kasei Kogyo Company, Japan), acryloyl chloride (98%, Tokyo Kasei Kogyo Company, Japan), tetrahydrofuran (THF) and diethyl ether (Yuanli Company, Tianjin, China), *N,N*‐dimethylaminopropyl acrylamide (DMAPA, 98%), β‐propiolactone (≥90%), 2,2‐diphenyl‐1‐picrylhydrazyl (DPPH), 2‐hydroxy‐2‐methyl‐1‐phenyl‐1‐propanone (IRGACURE 1173, 98%), and [2‐(methacryloyloxy) ethyl] dimethyl‐(3‐sulfopropyl) ammonium hydroxide (SBMA) were purchased from Sigma‐Aldrich and used as received. NAGA was synthesized according to our previous work.^23^ CBAA monomer was synthesized by the reaction of DMAPA and β‐propiolactone using a previously published method.^40^ All other chemicals and solvent were analytical reagents.

*Preparation of Poly(N‐acryloyl glycinamide‐co‐carboxybetaine acrylamide) (PNAGA‐PCBAA) Supramolecular Copolymer Hydrogels*: The PNAGA‐PCBAA hydrogels were prepared as the following procedures: An appropriate mass of NAGA and CBAA monomers with various mass ratios was dissolved in deionized water to form a homogenous solution, into which 2 wt% of photoinitiator IRGACURE 1173 (relative to the total mass of NAGA and CBAA) was added. The mixture was stirred vigorously and quickly injected into plastic rectangle molds or plastic cylinder molds. The polymerization was performed for 40 min in a crosslinked oven (XL‐1000 UV Crosslinker, Spectronics Corporation, NY, USA). After that, the resulting hydrogels were thoroughly washed with phosphate buffer saline (PBS, pH 7.4) to remove the impurities. In the same manner, poly(*N*‐acryloyl glycinamide) hydrogel was prepared. The obtained poly(*N*‐acryloyl glycinamide) homopolymer and copolymer hydrogels were named as PNAGA and PNAGA‐PCBAA‐b‐c, respectively (b represented the initial total monomer concentration and c was the mass ratio of NAGA to CBAA).

*Characterizations*: The ^1^H NMR spectra of CBAA monomer, PNAGA homopolymer, and PNAGA‐PCBAA copolymer were recorded by Varian INOVA spectrometer (500 MHz) using the D_2_O as a solvent. EWCs of the PNAGA and PNAGA‐PCBAA hydrogels were measured at room temperature by using a gravimetric method reported earlier.^41^ The refractive indexes of PNAGA and PNAGA‐PCBAA hydrogels were determined on an Abbe refractometer (WYA‐2W, Lumsail Industrial Inc., Shanghai, China). The visible light transmittance of the hydrogels was measured using a UV–Vis spectrophotometer (DU800, BeckmanCoulter Inc., Brea, CA) at a wavelength of 550 nm at 37 °C. Deionized water was used as a blank.

*Rheology Test of the Hydrogels*: Prior to measurement, all the hydrogel samples were immersed in PBS to reach swelling equilibrium. Then dynamic rheological experiments were carried out on a rheometer (HAKKE MASE ^Ш^, Germany) equipped with a plate with a diameter of 35 mm and a Peltier device for temperature control. During the rheological measurement, a solvent trap was used to prevent water evaporation. Oscillation amplitude sweeps from 0.1 to 1000 Pa at a frequency of 1 Hz were performed to determine the linear viscoelastic region. From the linear viscoelastic region, a stress of 10 Pa was chosen for the oscillatory rheology measurements.

The temperature sweep test was performed at a fixed frequency (1 Hz) and shear stress (σ = 10 Pa) over a range from 25 to 70 °C. Frequency sweep was conducted from 0.01 to 10 Hz at a 1% deformation at 37 °C. Shear viscosity was evaluated by increasing the shear rate from 0.01 to 500 s^−1^ at 37 °C. The alternate step strain sweeps of PNAGA‐PCBAA‐b‐4 (*b* = 10, 15, and 20) hydrogels were measured at a fixed frequency (1 Hz) at 37 °C. Amplitude oscillatory strains were switched from small strain (γ = 10%) to subsequent large strain (γ = 200%, 400%, and 600%) with 100 s for every strain interval.

*Measurement of Nonspecific Protein Adsorption*: Evaluation of the protein adsorption was carried out through a procedure adapted from the previous reports.^42^ In brief, the equilibrated hydrogel disks were immersed into 1 mL of BSA solution (2 mg mL^−1^) at 37 °C for 90 min. After the incubation, the disks were washed with PBS for three times to remove the loosely adsorbed proteins. Then the adsorbed protein was detached by ultrasound. The micro‐BCA protein assay kit (Boster Bio‐technology Co., Ltd.) was used to determine the concentration of the adsorbed protein according to the protocol. The amount of the protein was calculated from the concentration of the standard protein solution. Independent tests were performed in triplicate samples. In order to compare with other hydrophilic hydrogel's anti‐fouling behavior, we also prepared PNAGA‐PSBMA‐10‐4 hydrogel (10 represented the initial total monomer concentration (wt%) and 4 was the mass ratio of NAGA to SBMA). The amount of nonspecific protein adsorption on this hydrogel was determined with the same way as described above.

*Animal Test*: All the animal experiments were conducted in accordance with the guidelines of the Council for the Purpose of Control and Supervision of Experiments on Animals, Ministry of Public Health, China.

*Subcutaneous Implantation*: C57BL/6 six week old male mice weighing ≈20 g were used for subcutaneous implantation of the hydrogels, which were divided into two groups: PNAGA and PNAGA‐PCBAA‐10‐4 (three mice per group). Before implantation, the hydrogels were equilibrated in sterilized phosphate buffered saline (PBS) overnight, and transferred into a 1 mL sterile syringe equipped with a 22G needle. Then 200 µL of PNAGA hydrogel or PNAGA‐PCBAA‐10‐4 hydrogel was subcutaneously injected into two separate sites on the mice's dorsum. After implantation, postoperative care was taken to ensure the recovery of the mice. After 1 month postimplantation, the mice were euthanatized. The hydrogels and surrounding tissues were collected, fixed in 4% paraformaldehyde immediately, embedded in paraffin, sectioned, and stained with hematoxylin and eosin (H&E) or Masson's trichrome (M&T) for further histological analysis.

*Intravitreal Injection of Hydrogels and Postoperative Examinations*: Adult male rabbits weighing ≈2.5 kg were used for intravitreal injection of the hydrogels, which were divided into four groups: Normal, Control (sham‐operated group), PNAGA, and PNAGA‐PCBAA‐10‐4 (five rabbits per group). Before surgery, the rabbits were anesthetized via an intramuscular injection with xylazine hydrochloride (0.2 mL kg^−1^). All surgeries were performed in the left eyes of the rabbits except the sham‐operated group, and the right eye served as the surgical control. 0.5–1 mL of vitreous body was extracted and replaced with the hydrogel. To be specific, the equilibrated and sterilized PNAGA or PNAGA‐PCBAA‐10‐4 hydrogel was transferred into a 5 mL sterile syringe with a 22G needle and then injected into the vitreous cavity after vitrectomy surgery at room temperature. For sham‐operated group, the vitreous body of the right eye was extracted and injected immediately back into the cavity. At 8 and 16 weeks postoperation, detailed ophthalmic examinations were performed involving B‐scan ultrasonography, electroretinogram, fundus fluorescein angiography, and fundus photograph. The operative eyes were harvested and the eyeballs were immediately fixed with 4% paraformaldehyde. H&E stained method was used to investigate the eyeball's tissues.

*Statistical Analysis*: Data are expressed as the mean ± standard deviation (SD). Statistical analysis was performed using two population Student's *t*‐test to evaluated the protein adsorption with p* < 0.05.

## Conflict of Interest

The authors declare no conflict of interest.

## Supporting information

Supplementary

Supplementary

Supplementary
