# Supplementary material for: Antifouling Super Water Absorbent Supramolecular Polymer Hydrogel as an Artificial Vitreous Body
Source: Adv Sci (Weinh). 2018 Sep 25;5(11):1800711. doi: 10.1002/advs.201800711 (PMC6247043; doi:10.1002/advs.201800711)
Supplement: Supplementary file 1 — Supplementary [file ADVS-5-1800711-s002.pdf]

Copyright WILEY-VCH Verlag GmbH & Co. KGaA, 69469 Weinheim, Germany, 2018.

Supporting Information

for *Adv. Sci.,* DOI: 10.1002/advs.201800711

Antifouling Super Water Absorbent Supramolecular Polymer Hydrogel as an Artificial Vitreous Body

Hongbo Wang, Yuanhao Wu, Chunyan Cui, Jianhai Yang, and Wenguang Liu**

Supporting Information

Antifouling super water absorbent supramolecular polymer hydrogel as an artificial vitreous body

Hongbo Wang, Yuanhao Wu, Chunyan Cui, Jianhai Yang*, Wenguang Liu*

School of Materials Science and Engineering, Tianjin Key Laboratory of Composite and Functional Materials, Tianjin University, Tianjin 300350, China

*Corresponding authors E-mail: (wgliu@tju.edu.cn, yangjianhai615@163.com)

Cytotoxicity assay: Mouse fibroblast (L929) cells were seeded in a 96-well plate at 2×10^4 cells per well and incubated for 24 h at 37 ℃ in 5% CO_2 humidified atmosphere. Then the culture medium was removed and 200 μL extraction medium from the hydrogels was added to each well. After incubating for 24 h, the medium was replaced with 180 μL fresh complete medium and 20 μL MTT (5 mg mL^{-1} in PBS), and the plate was further incubated for 4 h. Then, all medium was removed and 150 μL per well DMSO was added, followed by shaking for 30 min. The absorbance of each well was measured at 570 nm on a ∑960 plate-reader (Meter-tech) with pure DMSO as a blank reading. Non-treated cell was used as a control and the relative cell viability (mean%±SD, *n*=5) was expressed as $Abs_{sample}/Abs_{control}\times100\%$.

Cell viability of the PNAGA and PNAGA-PCBAA hydrogels was also assessed by a Live-Dead staining kit (CFSE, Dojindo Laboratorise, Japan) according to manufacturer's guidelines.

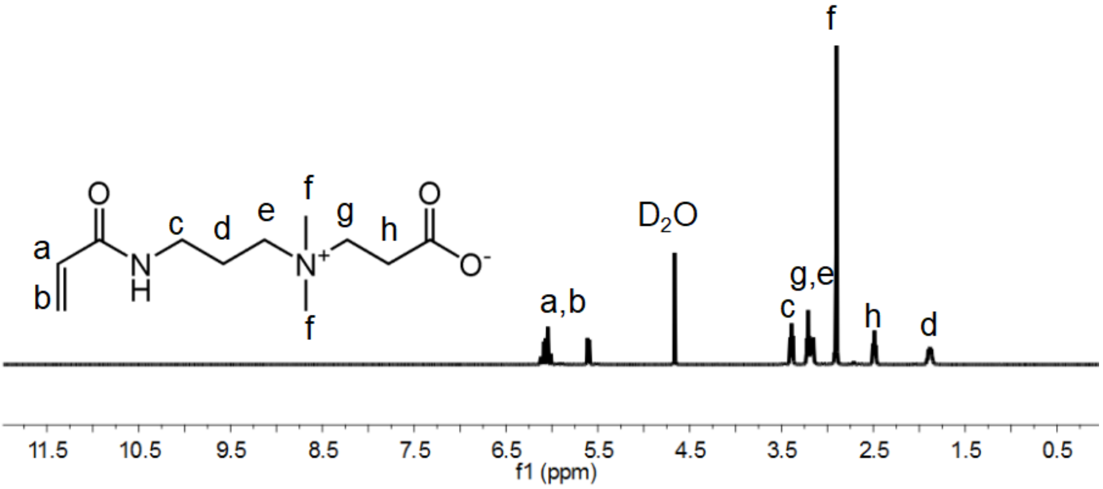

Figure S1. ^{1}H NMR spectrum of CBAA monomer.

The feature bands of CBAA present in the figure are in accordance with the literature data.[1] ^{1}H NMR of CBAA (500 MHz, D_2O) 1.88 (H_d, -CH_2-CH_2-CH_2-), 2.49 (H_h, -CH_2-CO-), 2.91 (H_f, N^+(CH_3)$_2$), 3.16-3.22 ($H_{g,e}$, -CH_2-N^+-CH_2-), 3.41 (H_c, -NH-CH_2-), 5.61-6.09 ($H_{a,b}$,CH_2=CH-CO-)

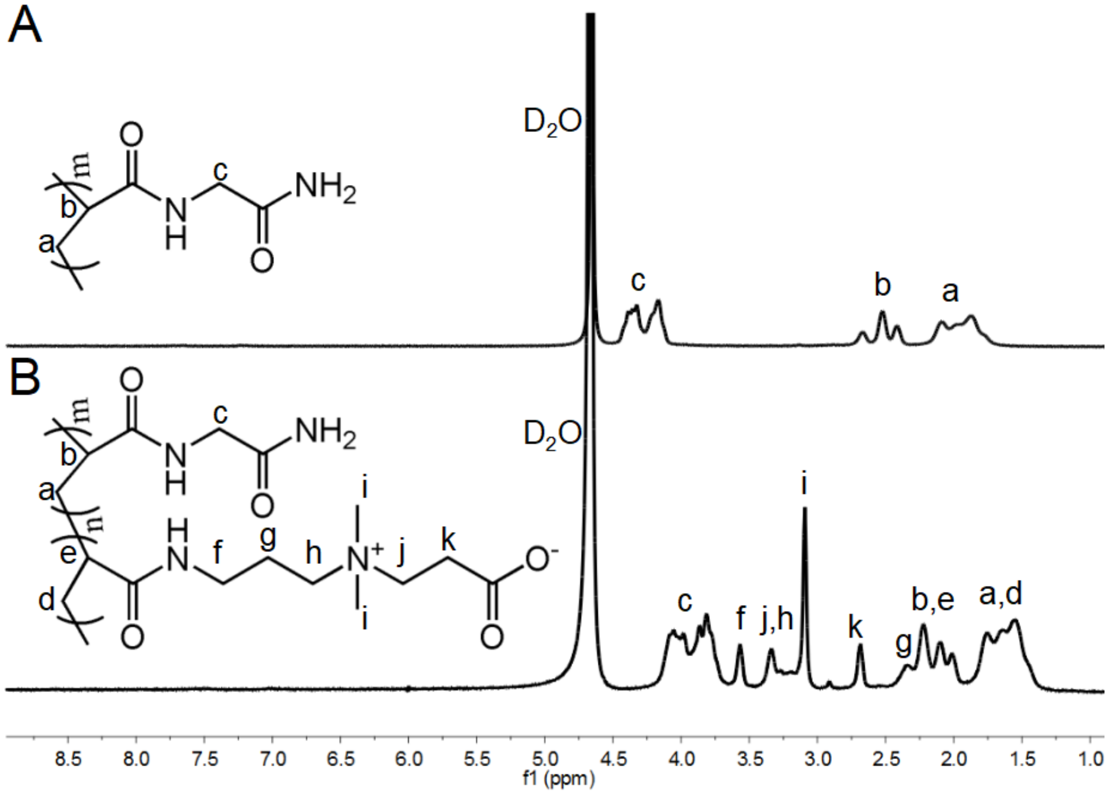

Figure S2. ^{1}H NMR spectra of (A) PNAGA homopolymer and (B) PNAGA-PCBAA copolymer. The feature bands of PNAGA polymer and PNAGA-PCBAA copolymer present in the figure are in accordance with the literature data.[2,3] ^{1}H NMR of PNAGA polymer (500 MHz, D_2O) 1.87-2.10 (H_a, -CH_2-CH-), 2.41-2.67 (H_b, -CH_2-CH-), 4.17-4.38 (H_c, -NH-CH_2-CO-); ^{1}H NMR of PNAGA-PCBAA copolymer (500 MHz, D_2O) 1.56-1.76 ($H_{a,d}$, -CH_2-CH-), 2.01-2.22 ($H_{b,e}$, -CH_2-CH-), 2.34 (H_g, -CH_2-CH_2-CH_2-), 2.68 (H_k, -CH_2-CO-), 3.09 (H_i, N^+(CH_3)$_2$), 3.34 ($H_{j,h}$, -CH_2-N^+-CH_2-), 3.57 (H_f, -NH-CH_2-), 3.82-4.07 (H_c, -NH-CH_2-CO-)

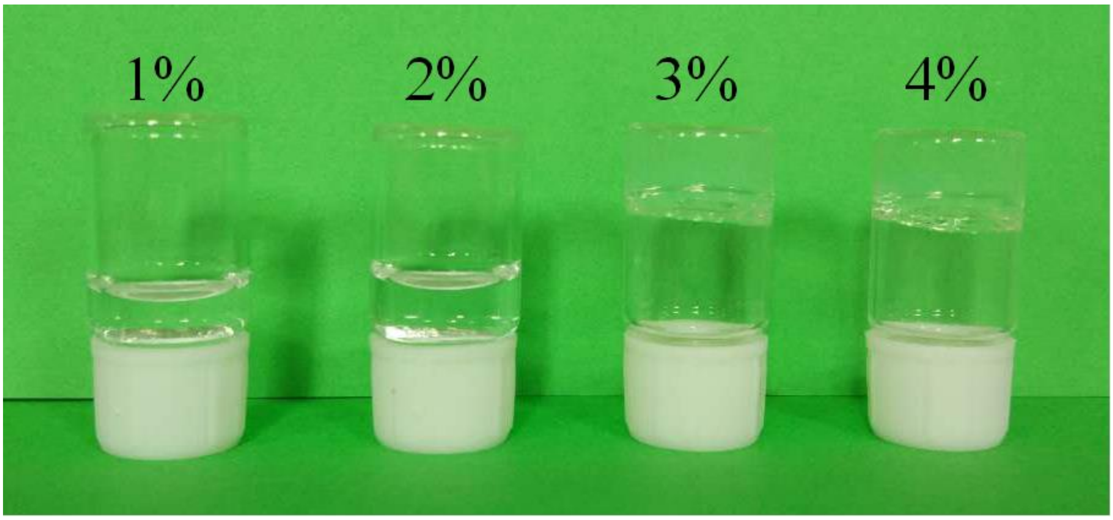

Figure S3. Photographs of PNAGA-PCBAA-b-4 polymerized at various monomer concentrations. Numbers in the image indicate the initial concentration of monomers by weight percentage. At 3 wt% monomer concentration, gelling occurred.

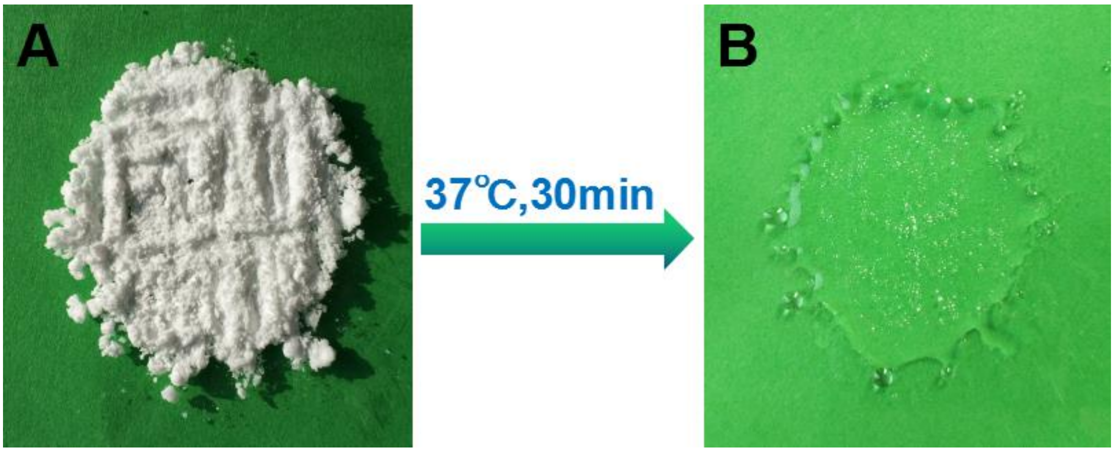

Figure S4. Image showing the deliquescence of the CBAA monomer. The CBAA monomer powder (A) absorbed moisture from air and turned into transparent liquid (B) within 30 min.

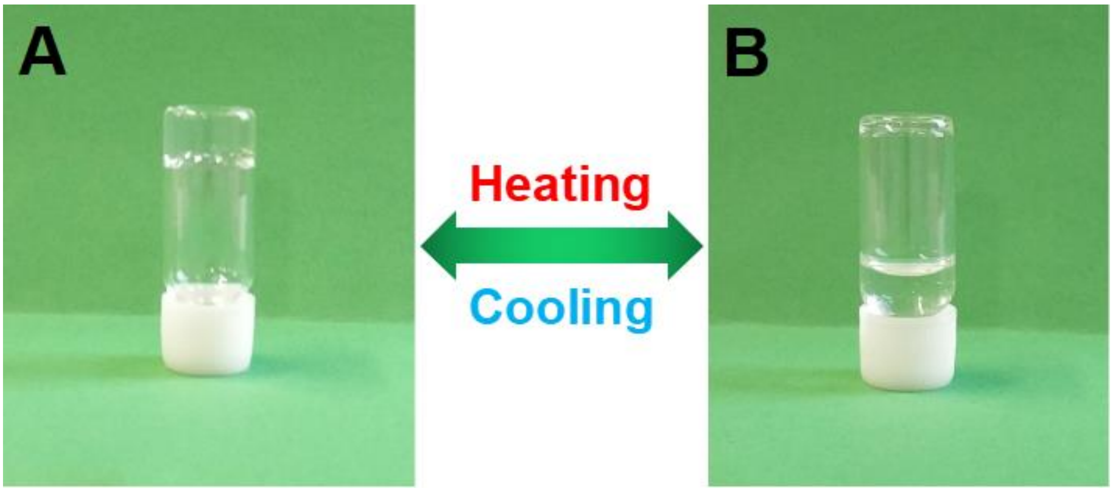

Figure S5. Photographs of test-tube inverting method demonstrating the reversible gel-sol and sol-gel transition of PNAGA-PCBAA-10-4 hydrogel during heating and cooling.

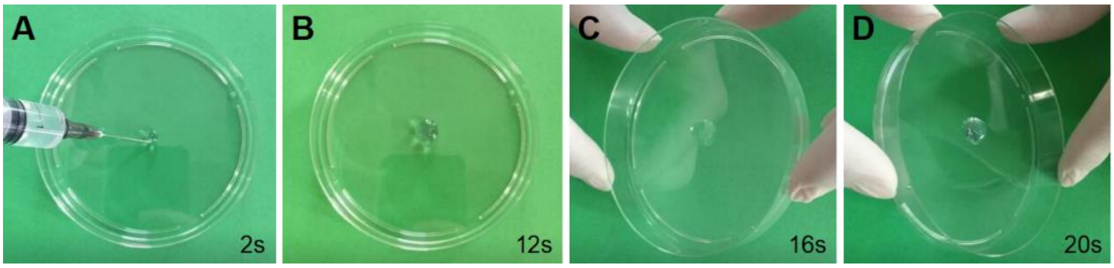

Figure S6. Injection process of PNAGA-PCBAA-10-4 hydrogel in vitro via a syringe with a 22G needle at various time points (A: 2s, B: 12s, C: 16s and D: 20s).

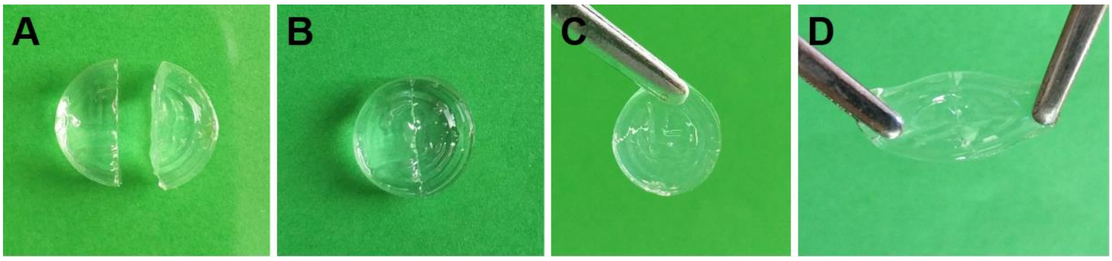

Figure S7. Exhibition of self-healing PNAGA-PCBAA-10-4 hydrogel.

A: The hydrogel disk was cut into two semicircles; B: The separate semicircles were brought into contact with each other; C: The hydrogel healed and could withstand its own weight; D: The self-healed hydrogel could withstand stretching.

Table S1. Equilibrium water content (EWC) and optical properties of the hydrogels.

Samples	Water content	Refractive index	Light transmittance before implantation	Light transmittance after implantation for 6 M
Human vitreous[4]	98-99%	1.3345-1.3348	>90%	-
PNAGA-PCBAA-10-4	98.4±0.6%	1.3354±0.0003	93.2±0.4%	92.9%±0.6%
PNAGA-PCBAA-15-4	90.1±0.9%	1.3426±0.0004	89.4±0.3%	-
PNAGA	94.7±0.7%	1.3572±0.0002	53.3±0.6%	51.1±0.8%

Table S2. Parameter comparison of various hydrogels as vitreous substitutes reported thus far.

Entry	Equilibrium water content (EWC, %)	Refractive index	Light transmittance (%)	Injection temperature (ºC)	Reaction method	Restorability of hydrogel	Data sources
PNAGA-PCBAA-10-4 hydrogel	98.4±0.6%	1.3354±0.0003	93.2±0.4%	RT	Not required	Yes	In this work (vitreous substitute group)
PNAGA	94.7±0.7%	1.3572±0.0002	53.3±0.6%	55 ºC	Not required	No	In this work (control group)
Human vitreous	98-99%	1.3345-1.3348	>90%	N/A	N/A	N/A	*Acta Biomater.* **2011**, *7*, 921.
Oligo-Tetra-PEG hydrogel	N/A	N/A	N/A	N/A	Thiol-maleimide (in situ)	N/A	*Nat. Biomed. Eng.* **2017**, *1*, 0044.
Thiolated-Gellan/Poly(MAM-co-MAA-co-BMAC) hydrogel	N/A	N/A	N/A	N/A	Redox-active disulfide bonds	N/A	*Macromolecules* **2016**, *49*, 4619.
Thiolated-Gellan/Poly(MAM-co-MAA-co-BMAC) hydrogel	N/A	1.3355	94%	45 ºC	Redox-active disulfide bonds (in situ)	N/A	*Acta Biomater.* **2016**, *43*, 327
PanaceaGel SPG-178 peptide gel	N/A	1.3339	96.7%	N/A	Self-assembling (in situ)	N/A	*Invest Ophthalmol Vis Sci.* **2017**, *58*, 4068.
Poly(acrylamide-acrylic acid) hydrogel	N/A	1.336	N/A	N/A	Redox-active disulfide bonds	N/A	*J. Bioact. Compat. Polym.* **2017**, *32*, 528.
Hyaluronic acid hydrogel	97%	1.341	N/A	N/A	Not required	N/A	*Graefes Arch. Clin. Exp. Ophthalmol* **2016**, *254*, 697.
Poly(MPDSA-co-AC) hydrogel	N/A	N/A	>90%	RT	Thiol-ene Michael addition reaction (in situ)	N/A	*J. Mater. Chem. B* **2015**, *3*, 1097
PVA hydrogel	98.1%	1.3361	93%	N/A	Not required	N/A	*Sci. Rep.* **2013**, *3*, 1838.
Oxidated-hyaluronic acid/adipic acid dihydrazide hydrogel	N/A	1.3442	N/A	4 ºC	Aldehyde-hydrazide condensation (in situ)	N/A	*J. Biomater. Sci. Polym. Ed.* **2011**, *22*, 1777
PEG-octadecyl groups gel	N/A	1.353	>90%	40 ºC	Self-assembling (in situ)	Yes	*Biomacromolecules*, **2011**, 12, 4011

N/A: The data are not available in the data sources; RT: room temperature

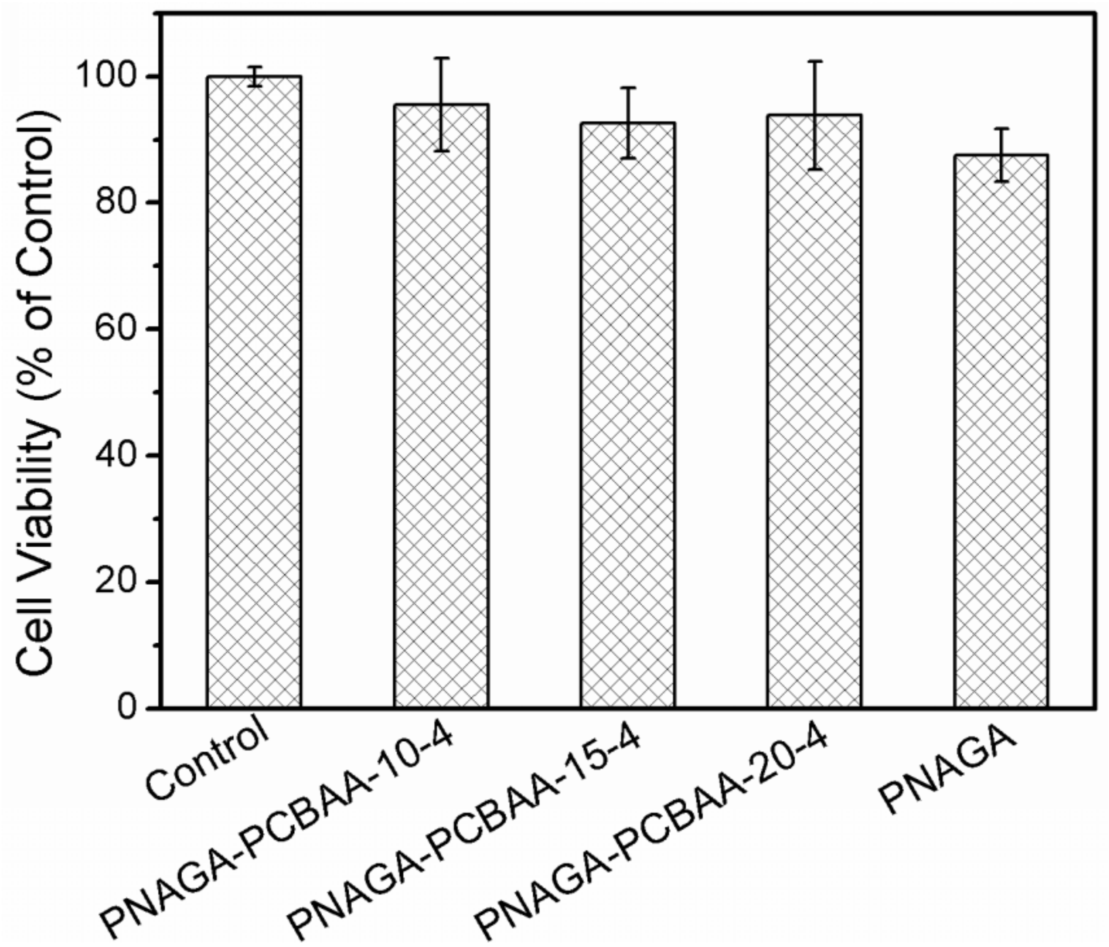

Figure S8. Cell viability of L929 cells cultured with the extraction medium of PNAGA-PCBAA hydrogels and PNAGA hydrogel.

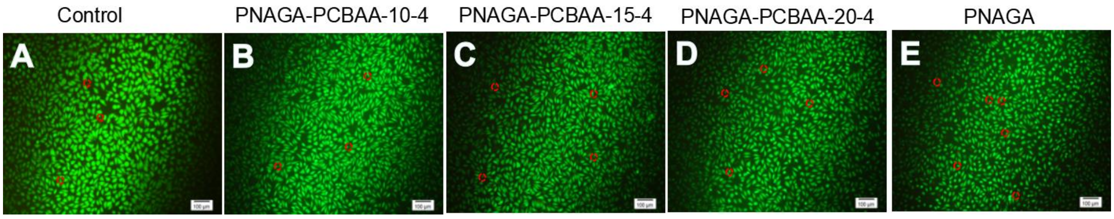

Figure S9. Fluorescence micrographs of L929 cells co-cultured with PNAGA-PCBAA hydrogels and PNAGA hydrogel. L929 cells were treated with calcein AM and propidium iodide. Green dots indicated the living cells and red dots indicated the dead cells.

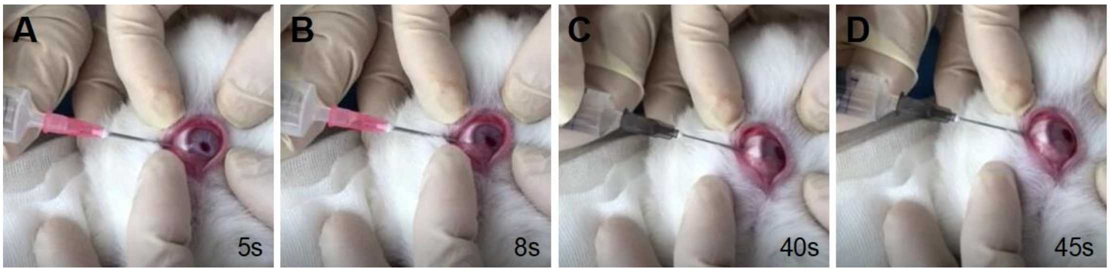

Figure S10. Vitrectomy and injection process of PNAGA-PCBAA-10-4 hydrogel at various time points. A and B: vitreous body was extracted via a syringe with a 18G needle. C and D: PNAGA-PCBAA-10-4 hydrogel was injected into vitreous cavity with a 22G needle syringe. The numbers on the photos were operation times.

Movie S1. Injection process of PNAGA-PCBAA-10-4 hydrogel *in vitro* via a syringe with a 22G needle.

Movie S2. Vitrectomy and injection process of PNAGA-PCBAA-10-4 hydrogel *in vivo*.

References

References

[1] Z. Zhang, H. Vaisocherova, G. Cheng, W. Yang, H. Xue, S. Jiang, Nonfouling behavior of polycarboxybetaine-grafted surfaces: structural and environmental effects, *Biomacromolecules* **2008**, *9*, 2686.

[2] H. Wang, H. Zhu, W. Fu, Y. Zhang, B. Xu, F. Gao, Z. Cao, W. Liu, A high strength self-healable antibacterial and anti-inflammatory supramolecular polymer hydrogel, *Macromol. Rapid Commun.* **2017**, *38*, 1600695.

[3] F. Sun, K. Wu, H. C. Hung, P. Zhang, X. Che, J. Smith, X. Lin, B. Li, P. Jain, Q. Yu, S. Jiang, Paper sensor coated with a poly(carboxybetaine)-multiple DOPA conjugate via dip-coating for biosensing in complex media, *Anal. Chem.* **2017**, *89*, 10999.

[4] F. Baino, Towards an ideal biomaterial for vitreous replacement: Historical overview and future trends, *Acta Biomater* **2011**, *7*, 921.
